# Biology, temperature thresholds, and degree-day requirements for development of the cucumber moth, *Diaphania indica* , under laboratory conditions

**DOI:** 10.1093/jis/14.1.61

**Published:** 2014-01-01

**Authors:** Sareh Hosseinzade, Hamzeh Izadi, Pyman Namvar, Mohamad Amin Samih

**Affiliations:** 1 Department of Plant Protection, Vali-e-Asr University of Rafsanjan, Iran; 2 Plant Pests and Diseases Research Center, Jiroft, Iran

**Keywords:** development period, greenhouse pest

## Abstract

The cucumber moth, *Diaphania indica* (Saunders) (Lepidoptera: Pyralidae), is a tropical and sub-tropical cucurbits pest and a key greenhouse pest in the Jiroft region of Iran. In this study, the effect of different temperatures on the development of this pest was investigated on cucumber, *Cucumis sativus* L. (Cucurbitales: Cucurbitaceae), leaves in a growth chamber at various constant temperatures (20, 25, 30, and 35ºC). The results indicated that the development period from egg to adult death at the decreased with increasing temperature. Mortality was greatest at 35ºC. Based on a linear model, the highest and lowest temperature thresholds were recorded for male insects and pupal stage as 16ºC and 9.04ºC with thermal constants of 100 and 144.92 degree days, respectively.

## Introduction

The cucumber moth, *Diaphania indica* (Saunders) (Lepidoptera: Pyralidae), is a polyphagous pest and is particularly serious on cucurbits. Larvae mainly attack leaves, but also infest flowers and fruits, and cause considerable yield loss during outbreak. It is also known as the cotton caterpillar and pumpkin caterpillar. This pest infests cucumber ( *Cucumis sativus* L.), melon ( *C. melo* L.), gherkin ( *C. sativus* L.), bottle gourd ( *Lagenaria siceraria* Molina), bitter gourd ( *Momordica charantia* L.), snake gourd ( *Trichosanthes an- guina* L.), Luffa ( *Luffa* aegyptiaca Mill.), little cucumber ( *Melothria* spp), cotton ( *Gossypium hirsutum* L.), and more. The preferred hosts of the larvae of this pest are cucumber ( *Cucumis sativus* L.), gourd ( *Lagenaria siceraria* Molina), watermelon ( *Citrullus lanatus* Thunb), oriental melon ( *Cucumis melo var. makuwa* L.), wax gourd ( *Benincasa hispida* Thunb), melon ( *Cucumis melo* L.) star cucumber ( *Sic- yos angulatus* L.), sponge cucumber ( *Luffa cylindrica* L.), cotton ( *Gossypium indicum* L.), bitter gourd ( *Momordica charantia* L.), little gourd ( *Coccinia indica* L.), and pointed gourd ( *Trichosanthes dioica* Roxb) ( Tripathi and Pandy 1973 ; Pandy 1977 ; Clavijo et al. 1995 ; Ravi et al. 1998 ; Capinera 2001 ; Choi et al. 2003 ). This species is mostly distributed in Pakistan, India, Japan, Pacific Islands, Australia, Africa, and South America ( Peter and David 1991 ; Capinera 2001 ).

Various studies are available on the biology of *D. indica* all over the world. The change in population of this pest was studied in India on pumpkin, *Coccinia grandis* L. ( Peter and David 1992 ). The field biology of this pest was studied in South Korea ( Choi et al. 2003 ), Yemen ( Angood and Angood 1979 ), and China ( Ke et al. 1988 ). Various aspects of the biology and natural enemies of this pest were studied by Ganehiarachchi (1997) . The effects of different temperatures on development and reproduction of the pest were investigated by Kinjo and Arakaki (2002) . Shin et al. (2002) and Korgaonkar et al. (2004) investigated the effect of host plants on the biology of the pest.

Data on the biology of *D. indica* in Iran are scarce. This study was conducted in order to understand the biology of the pest in Iran at different temperatures and to determine the optimal temperature for the development of this pest.

## Materials and Methods

### Rearing of *D. indica*

Larvae of *D. indica* were collected from cucumbers grown in greenhouses located in Jiroft, Iran, in November 2010. They were reared on cucumber, *Cucumis sativus* L. (Cu- curbitales: Cucurbitaceae), leaves (Tunca variety) at 25 ± 1ºC and 65 ± 5% RH with a 16:8 L:D photoperiod until pupation. Pupae were sexed and kept in separate plastic Petri dishes until adult emergence. One-day-old pairs of virgin females and males were confined to plastic containers (8 × 10 cm) for mating and were provided with a piece of cucumber leaf as an oviposition substrate and cotton soaked in honey diluted with water (1:10) as food. Freshly laid eggs on the cucumber leaves were counted and transferred into plastic Petri dishes.

### Biological study of *D. indica* at different temperatures

To study the biology of *D. indica* , 60 newly deposited (one-day-old) eggs in three replications were separately placed in Petri dishes and kept at four constant temperatures (20, 25, 30 and 35°C) at 65 ± 5% RH and with a 16:8 L:D until eclosion. Newly-hatched larvae were individually reared in separate plastic Petri dishes on a piece of fresh cucumber leaf (the Petri dishes and cucumber leaves were changed every two days). The bottom of each Petri dish was lined with slightly moistened filter paper to prevent desiccation until pupation. Pupae were sexed (in each temperature, the sex ratio was assessed) and kept in separate plastic Petri dishes until adult emergence to determine sex ratio at each temperature. Pairs (30–50) of virgin females and males (one-day-old) were confined in plastic containers (8 × 10 cm) for mating. Male and female moths were mated in the first night for 24 hr, and then each mated female was released separately into a plastic container (8 × 10 cm). To facilitate ventilation, a 3 cm diameter opening was cut in the top of the plastic container and covered with nylon mesh. During the reproduction period, a cotton ball soaked with 10% honey-water solution was placed in the container for feeding. Females were transferred daily to new plastic containers with a piece of fresh leaf and honey. For the males, honey solution was added to the cotton each day. Plastic containers and cotton were replaced at two or three day intervals. Developmental stages were checked daily with a stereomicroscope (Olympus, www.olympus-global.com ), and developmental period and mortality of eggs, larvae, pupae, and adults were recorded. The larval instars were recognized by molting.

### Thermal requirements for development of *D. indica*

The results of rearing *D. indica* at different constant temperatures were used to calculate the developmental time for all stages *.* For calculation of developmental rate of the different stages at the various temperatures, 1/insect developmental period was used. The linear model ( Campbell et al. 1974 ) was used to estimate the lower temperature threshold ( *T* 0) and the thermal constant ( *K* ) for all stages of *D. indica.* The model of Campbell et al. (1974) is based on the linear regression equation, *r* ( *T* ) *= a + bT* , where *r* ( *T* ) is the rate of development and *T* is the temperature (°C). The parameter *a* is the intercept and *b* the slope of the straight line. The lower temperature threshold is calculated as *T* 0 = – *a* / *b* and the thermal constant as *K* = 1/ *b* .

### Statistical analysis

The normality and homogeneity of data were analyzed using oneway ANOVA (Minitab 14.0, www.minitab.com ), and the differences between means were determined using the least significant difference test (Duncan’s multiple range test) with the *P* -value set at 0.05 (SPSS 16.0, IBM, www.ibm.com ).

## Results

### Effect of temperature on biological parameters of *D. indica*

The results showed that temperature had a significant effect on the developmental time of different immature and adult stages, i.e., egg, 1st instar larvae, 2nd instar larvae, 3rd instar larvae, 4th instar larvae, 5th instar larvae, prepupa, pupa, adult, male adult, female adult, total developmental period of larvae, egg to adult emergence (developmental time), and egg to adult death (total). In the 1st, 2nd, and 5th larval instars, prepupae, and total larval period, developmental period increased slightly as temperature moved from 25 to 30ºC, and then decreased at 35ºC ( Table 1 ). Our results are in agreement with the results of Peter and David (1992) . However, Kinjo and Arakaki (2002) found that the development of this pest slowed down at high temperatures, and the development time at 35ºC was significantly greater than 30ºC. At 30ºC, the developmental time from egg to adult emergence in this study (19.91 days) was close to the 18.2 days reported in a Japanese population of *D. indica* ( Kinjo and Arakaki 2002 ), and lower than the 23.4 days re- reported in an Indian population ( Peter and David 1992 ). The temperature for shortest developmental time in this study (35ºC) was greater than the Japanese population (30ºC) and lower than Indian population (40ºC). The variation among these temperatures may be due to the effect of host plant on developmental time of *D. indica.*Ravi et al. (1998) studied the effect of several species of cucurbits on the development of *D. indica* , and Shin et al. (2002) investigated the effect of five different host plants (cucumber, pumpkin, watermelon, oriental melon, and melon) on the biological properties of this pest, and both concluded that the host type had a significant effect on development and reproduction of this pest. Differences in the developmental time of *D. indica* in different regions could also be attributed to geographical race, type of host, and laboratory conditions. Sex ratio increased proportionally with increases in tem- temperature from 20 to 35ºC and was greatest at 35ºC, but the increase was not significant ( Table 1 ).

**Table 1.**
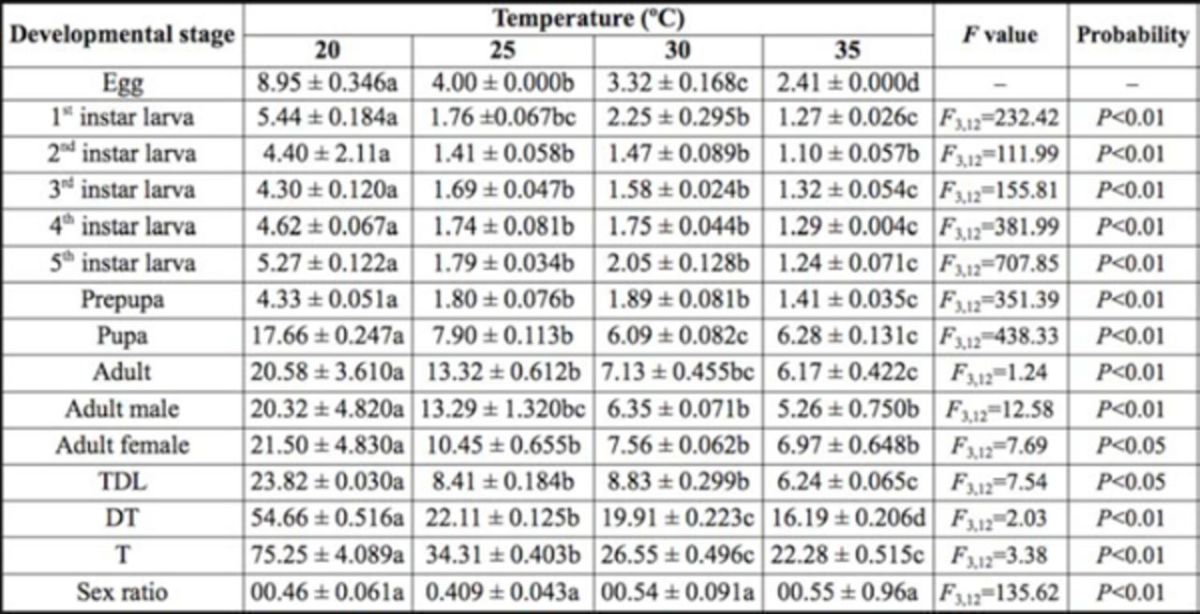
Mean (± SE) developmental period of *Diaphania indica* at four different temperatures.

Different letters in rows indicate a significant difference at the 5% level according to Duncan’s multiple range test. TDL = total developmental period of larva; DT = developmental time (egg to adult emergence), T = total (egg to adult death).

### Effect of temperature on mortality of various developmental stages of *D. indica*

The temperature did not have a significant effect on the mortality of immature stages, i.e., egg, 1st instar larvae, 2nd instar larvae, 3rd instar larvae, 4th instar larvae, 5th instar larvae, prepupa, and pupa ( Table 2 ). However, adult mortality was significantly affected by different temperatures. Maximum adult mortality was recorded at 30ºC ( Table 2 ).

**Table 2.**
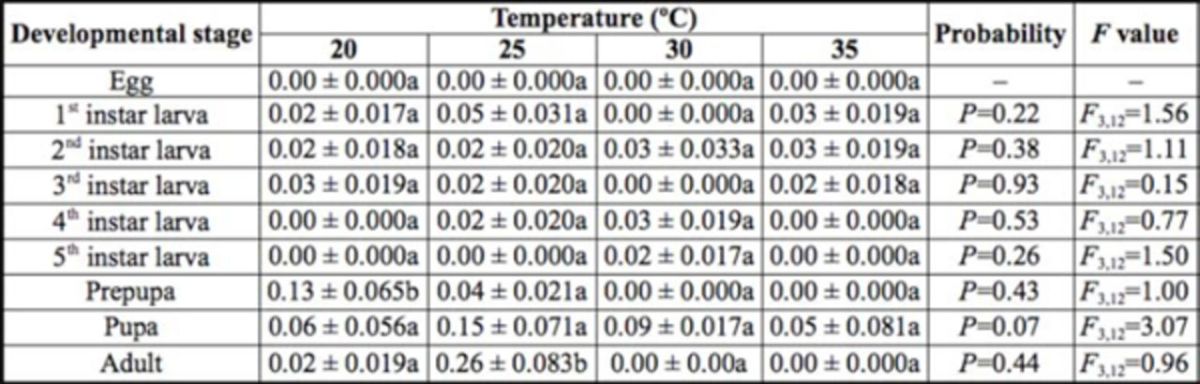
Mean (± SE) mortality *of Diaphania indica* at four different temperatures.

Different letters in rows indicate a significant difference at the 5% level according to Duncan’s multiple range tests.

### Thermal requirements for development of *D. indica*

The effect of different temperatures on the developmental rate of *D. indica* for all stages is shown in Table 3 . The observed pupal developmental time at 35°C was longer than predicted by the linear relationship between developmental rate and temperature. Thus, the data for this temperature were not included when the linear regression equation was used to obtain the lower temperature threshold and thermal constant. The results are in agreement with Shimizu (2000) , who reported that the lower temperature threshold and the thermal constant of egg to adult emergence of D. *indica* were 12.3ºC and 357.0 DD, respectively, on artificial diet. Kinjo and Arakaki (2002) recorded the highest lower temperature threshold for pupa (14.9ºC) and the lowest lower temperature threshold for larvae (12.0ºC) with thermal constants of 17.24 and 82.6 DD, respectively. In that study, the lower temperature threshold and the thermal constant for development of the egg to adult emergence were determined to be 13.5°C and 294.1 DD, respectively. In another investigation, the thermal constant and the lower temperature threshold of egg to adult emergence were determined to be 12.05ºC and 454.55 DD, respectively ( Peter and David 1992 ). Due to large climate changes between different regions of *D. indica* distribution, it is likely that the local populations or strains have adapted to these conditions. In conclusion, the results of our study suggest that 35ºC, which was correlated with the lowest developmental time and the highest sex ratio, is the best temperature for rearing of *D. indica* in the region. These results will provide insight into improving pest control.

**Table 3.**
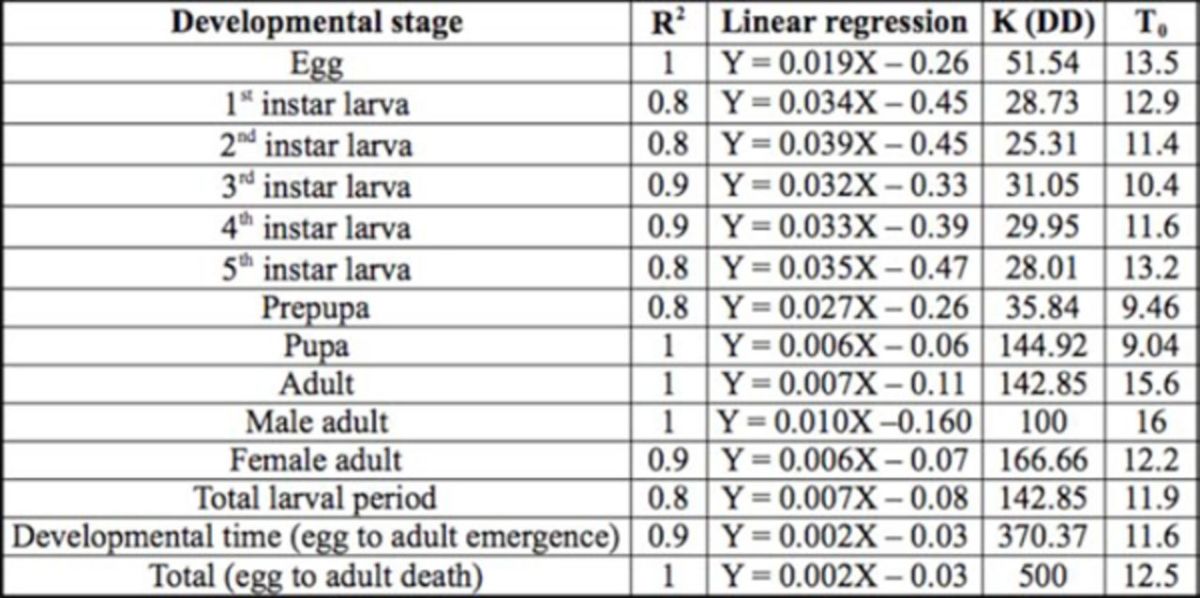
The lower developmental threshold (T0) and thermal constant K (DD) of *Diaphania indica* at four different temperatures.

DD = degree days; DT = developmental time (egg to adult emergence), T = total (egg to adult death).
